# Exposure to 15% oxygen *in vivo* up‐regulates cardioprotective SUR2A without affecting ERK1/2 and AKT: a crucial role for AMPK

**DOI:** 10.1111/jcmm.13064

**Published:** 2017-01-25

**Authors:** Khaja Shameem Mohammed Abdul, Sofija Jovanović, Aleksandar Jovanović

**Affiliations:** ^1^ Division of Molecular & Clinical MedicineNinewells Hospital & Medical SchoolUniversity of DundeeDundeeUK

**Keywords:** hypoxia, oxygen, SUR2A, heart, AMPK

## Abstract

SUR2A is an ‘atypical’ ABC protein that forms sarcolemmal ATP‐sensitive K^+^ (K_ATP_) channels by binding to inward rectifier Kir6.2. Manipulation with SUR2A levels has been suggested to be a promising therapeutic strategy against ischaemic heart diseases and other diseases where increased heart resistance to stress is beneficial. Some years ago, it has been reported that high‐altitude residents have lower mortality rates for ischaemic heart disease. The purpose of this study was to determine whether SUR2A is regulated by mild‐to‐severe hypoxic conditions (15% oxygen; oxygen tension equivalent to 3000 m above sea level) and elucidate the underlying mechanism. Mice were exposed to either to 21% (control) or 15% concentration of oxygen for 24 hrs. Twenty‐four hours long exposure to 15% oxygen decreased partial pressure of O2 (PO_2_), but did not affect blood CO_2_ (PCO_2_), haematocrit nor levels of ATP, lactate and NAD+/NADH in the heart. Cardiac SUR2A levels were significantly increased while Kir6.2 levels were not affected. Hypoxia did not induce phosphorylation of extracellular signal‐regulated kinases (ERK1/2) or protein kinase B (Akt), but triggered phosphorylation of AMP activated protein kinase (AMPK). AICAR, an activator of AMPK, increased the level of SUR2A in H9c2 cells. We conclude that oxygen increases SUR2A level by activating AMPK. This is the first account of AMPK‐mediated regulation of SUR2A.

## Introduction

ATP‐sensitive K^+^ (K_ATP_) channels link intracellular metabolic conditions with cellular membrane excitability, and they play a significant role in regulating resistance to stress in different cell types 1, 2, 3. The sulfonylurea receptor 2A (SUR2A) is an ‘atypical’ ABC transporter that, although possessing a structure of an ABC protein, it does not mediate transport, but binds to the inward rectifier Kir6.2 to form sarcolemmal K_ATP_ channels 4. Recent studies showed the involvement of SUR2A in regulation of myocardial resistance to metabolic and oxidative stress 5, 6, 7, 8, 9 and to cardiac ageing 10, 11. It has been shown that increased level of SUR2A (*i*) protects myocardium against ischaemia‐reperfusion and other types of oxidative stress 5, 6, 7, 8, 9, (*ii*) increases physical endurance 7, (*iii*) counteracts ageing‐induced increase in myocardial susceptibility to metabolic stress and decrease in physical endurance 10 and (*iv*) reprogrammes embryonic cardiomyocytes towards less differentiated stem‐like cells 12.

Manipulation of SUR2A levels has been suggested to be a promising therapeutic strategy against ischaemic heart diseases and other diseases where increased heart resistance to stress is beneficial 13, 14, 15. Some years ago, it has been reported that high‐altitude residents have lower mortality rates due to ischaemic heart disease 15, 16, 17, 18. In agreement with these early studies were later findings that exposure to moderate hypoxia confers cardioprotection to rats 19, 20. Under *in vitro* conditions, it has been shown that SUR2A levels are more sensitive to changes in oxygen tension than other proteins 21. Recent *in vivo* studies demonstrated that exposure to subhypoxia (20% oxygen; equivalent to 350 m above sea level), mild hypoxia (18% oxygen; equivalent to 1200 m above sea level) or severe hypoxia (13% oxygen; equivalent to 3600 m above sea level) increases level of SUR2A and myocardial resistance to ischaemia‐reperfusion 8, 9, 22. It has been shown that different oxygen tensions activate different signalling cascades that have up‐regulation of SUR2A as common mechanism. Specifically, 20% oxygen activates ERK1/2, 18% oxygen activates PI3K/Akt and 13% oxygen does not activate any of tested kinases 8, 9, 22. About 15% oxygen corresponds to oxygen tension found at ~3000 m above sea level. It is yet unknown whether this level of hypoxia would regulate SUR2A and what the signalling cascade would be. Therefore, in this study we have tested *in vivo* the impact of 24 hrs exposure to 15% oxygen on various biochemical and molecular parameters in experimental mice. Not only that we have found that this concentration of oxygen increased level of SUR2A but that this is due to the activation of AMPK independently from ERK1/2 and PI3K/Akt.

## Materials and methods

### Mice and *in vivo* exposure to hypoxia

C57BL/6J male mice (6–8 weeks old) were exposed to either ambient oxygen (detected to be 21%) or 15% oxygen (normobaric hypoxia) using integral Animal Hypoxia Chamber System; oxygen levels were controlled by ProOx Model 110 version 2.2 (Biospherix, Lacona, NY, USA). Mice, in groups of 5 (comment requiring response), were placed in a plexiglass chamber for 24 hrs in either 21% or 15% oxygen, under continuous monitoring of oxygen level. Twenty‐four hours was the time of exposure as we have shown previously that within this time frame, subhypoxia and different degrees of *in vitro* and *in vivo* hypoxia regulate the level of SUR2A 8, 9, 21, 22. Additionally, other agents regulating SUR2A expression also act within 24 hrs 23, 24. All manipulation with animals including hearts harvesting were performed inside the chamber. For heart harvesting, mice were killed using a schedule 1 procedure of cervical dislocation. The experiments have been carried out under the authority of Project Licence 70/7796.

### H9c2 cells

*In vitro* experiments were performed on rat embryonic heart‐derived H9c2 cells (ECACC, Salisbury, UK). Cells were cultured in DMEM medium containing 2 mM glutamine and 10% FCS at 37°C and 5% CO_2_. In particular experiments, AICAR (1 mM in DMSO) was added into the culture media and DMSO‐containing samples were considered as control (1.3% was final DMSO concentration). The cultures were then left for a 24‐hrs incubation period before experimentation.

### Blood gas and haematocrit analysis

Blood gas (PO_2_ and PCO_2_) and haematocrit (HCT) were measured in blood (500–700 μl) taken directly from the heart of experimental animals using pre‐heparinized (1000 IU/ml) syringes and Rapidlab 348EX blood Gas System (Siemens, Frimley, UK).

### Western blotting

For Western blotting, hearts/H9c2 cells were harvested and snap‐frozen and homogenized in lysis buffer (50 mM Tris–HCl, pH 7.5; 1 mM EDTA; 1 mM EGTA; 1% (w/v) Triton X‐100; 0.1% (v/v)‐mercaptoethanol; 1 mM sodium orthovanadate; 50 mM sodium fluoride; 5 mM sodium pyrophosphate; 1 μM microcystin‐LR; and one tablet of ‘complete’ proteinase inhibitor per 50 ml of buffer). Specifically, a 10‐fold mass excess of ice‐cold lysis buffer was added to the powdered tissue/cells, briefly vortexed and then centrifuged at 4°C for 10 min. at 13,000 ×*g* to remove insoluble material. The supernatant was snap‐frozen in aliquots in liquid nitrogen and stored at −80°C. Protein concentration was determined by the Bradford assay. From each sample, 20 μg of protein was subjected to SDS/PAGE and transferred to nitrocellulose membranes. For all blots, the nitrocellulose membranes were incubated at 4°C for 16 hrs using antibodies against SUR2A, Kir6.2 (both from Santa Cruz Biotechnology, Heidelberg, Germany), Akt, AMPK, ERK1/2, phospho‐Akt antibodies (Thr308 and Ser473), phosphor‐AMPK and phospho‐ERK1/2 (all from Millipore, Watford, UK). All antibodies were applied in 1:1000 dilution. The blots were incubated in 50 mM Tris/HCl, pH 7.5; 0.15 M NaCl; and 0.2% (v/v) Tween containing 5% (by mass) skimmed milk. Detection of total or phosphorylated protein was performed using horse radish peroxidase‐conjugated secondary antibodies (Pierce, Rockford, IL, USA) and enhanced chemiluminescence reagent (Upstate, Dundee, UK). The band intensities were analysed using QUANTISCAN software (Biosoft, Cambridge, UK). All blots have been performed, at least, in triplicates.

### Measurement of ATP in the heart

ATP concentration in heart tissue was measured using luciferase‐based ATP determination kit (Invitrogen, Paisley, UK) according to the manufacturer instructions. Luminescence was measured at 560 nm using microplate reader/multidetection reader (SpectraMax M2; Molecular Devices, Wokingham, UK).

### Measurement of NAD/NADH in the heart

NAD/NADH was measured in heart tissue using NAD/NADH kit (Abcam, Cambridge, UK) according to the manufacturer's instruction. Absorbance was measured at 450 nm using microplate reader/multidetection reader (SpectraMax M2; Molecular Devices). Total NAD (NADt) and NADH were estimated directly while the value of NAD^+^ was estimated by subtracting NADH from NADt.

### Measurement of lactate in the heart

Lactate was measured in heart tissue lysates using ADVIA Chemistry Lactate Enzymatic Assay and ADVIA Chemistry System 1200 (Siemens). Lactate is oxidized by lactate oxidase to pyruvate and hydrogen peroxide, and it was measured by the formation of dye from hydrogen peroxide and a chromogen in the presence of a peroxidase according to manufacturer instructions. Absorbance was measured at 545/694 nm.

### Statistical analysis

Data are presented as mean ± S.E.M, with *n* representing the number of analysed mice or independent experiments when H9c2 cells were used. Mean values for all measurements were compared by Student's *t*‐test or Mann–Whitney rank sum test where appropriate using SigmaStat program (Jandel Scientific, Chicago, IL, USA). *P* < 0.05 was considered statistically significant.

## Results

### 24‐hrs‐long exposure to 15% oxygen up‐regulates SUR2A in the heart

**Figure 1:**
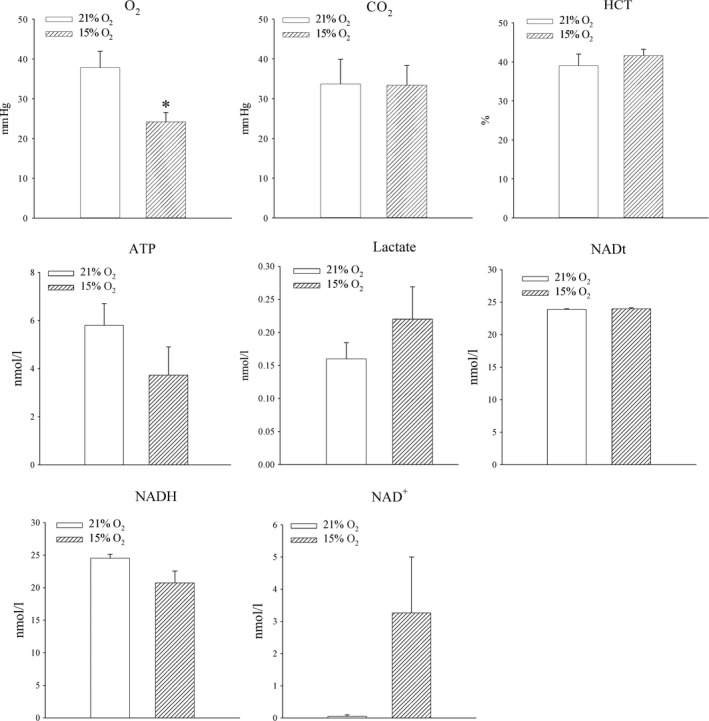
The effect of systemic exposure to 15% oxygen on different parameters in blood and myocardial tissue. Bar graphs depicting PO_2_ (O_2_), PCO_2_ (CO2) and haematocrit (HCT) in the blood as well as ATP (ATP), lactate (Lactate), total NAD (NADt), NADH (NADH) and NAD+ (NAD+) in the myocardial tissue. Each bar is a mean ± S.E.M. (*n* = 3–5). *P < 0.05.

**Figure 2:**
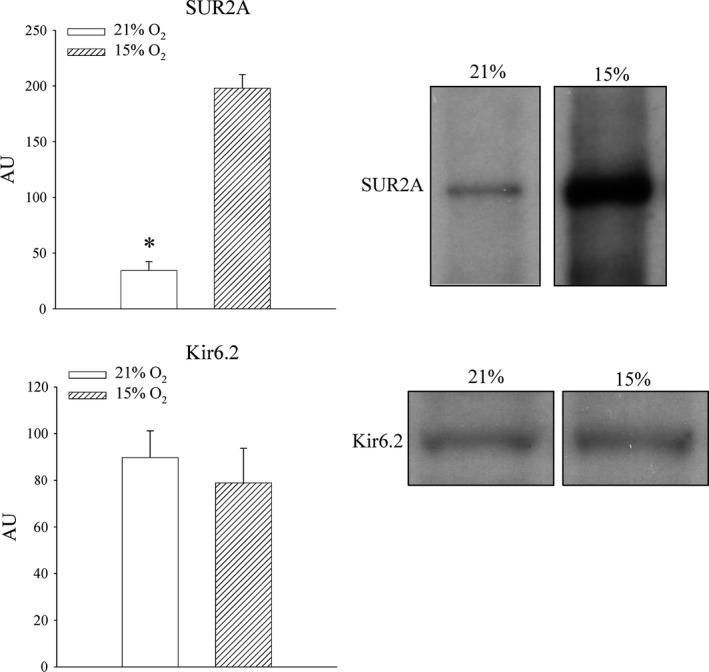
The effect of systemic exposure to 15% oxygen on SUR2A and Kir6.2 levels in myocardial tissue. Original Western blots and corresponding graphs under labelled conditions. Each bar is a mean ± S.E.M. (*n* = 3–5). *P < 0.05. AU: arbitrary units.

Mice were exposed to either atmospheric oxygen that was measured to be 21% (Dundee is situated at 74 m above the sea level) or to mild‐to‐severe hypoxic conditions (15% oxygen). Oxygen tension in the blood was decreased in mice exposed to 15% oxygen (PO_2_ in the blood was 37.8 ± 5.8 mmHg in mice exposed to 21% oxygen and 24.2 ± 2.4 mmHg in mice exposed to 15% oxygen, *n* = 4–5, *P* = 0.073; Fig. 1). No differences were observed between blood levels of CO_2_ (PCO_2_ in the blood was 33.7 ± 6.2 mmHg in mice exposed to 21% oxygen and 33.4 ± 4.9 mmHg in mice exposed to 15% oxygen, *n* = 4 for each, *P* = 0.972; Fig. 1), and HCT did not differ between experimental groups (HCT was 39.0 ± 3.0% in mice exposed to 21% oxygen and 41.6 ± 1.6% in mice exposed to 15% oxygen, *n* = 5 for each, *P* = 0.434; Fig. 1). The level of ATP in heart tissue was lower in mice exposed to 15% oxygen, but the difference was not statistically significant (5.8 ± 0.9 mmol/l in mice exposed to 21% oxygen and 3.7 ± 1.2 mmol/l in mice exposed to 15% oxygen, *P* = 0.200; *n* = 5 for each; Fig. 1). Lactate was also increased by 15% oxygen exposure, but, again, difference was not statistically significant (0.16 ± 0.02 mmol/l in mice exposed to 21% and 0.22 ± 0.05 mmol/l in mice exposed 15% oxygen, *P* = 0.305, *n* = 5 for each; Fig. 1). Exposure to 15% oxygen did not significantly altered total levels of NAD (23.9 ± 0.1 nml/l for 21% oxygen and 24.0 ± 0.2 nmol/l for 15% oxygen, *P* = 0.663, *n* = 3–4; Fig. 1), nor levels of NADH (24.5 ± 0.6 nmol/l for 21% oxygen and 20.7 ± 1.8 nmol/l for 15% oxygen, *P* = 0.146, *n* = 3–4; Fig. 1) or NAD^+^ (0.05 ± 0.05 nmol/l for 21% oxygen and 3.3 ± 1.7 nmol/l for 15% oxygen, *P* = 0.180; Fig. 1). Exposure to 15% oxygen significantly increased level of SUR2A in mouse hearts (Fig. 2; signal intensity was 34.4 ± 8.1 AU (arbitrary unit) in mice exposed to 21% oxygen and 198.1 ± 12.0 AU in mice exposed to 15% oxygen, *n* = 4–5, *P* < 0.001). On the other hand, the level of Kir6.2 was not affected (Fig. 2; signal intensity was 89.7 ± 11.5 AU in mice exposed to 21% oxygen and 78.8 ± 14.9 AU in mice exposed to 15% oxygen, *n* = 3, *P* = 0.594).

### 24‐hrs‐long exposure to 15% oxygen does not phosphorylate Akt or ERK1/2 in the heart

**Figure 3:**
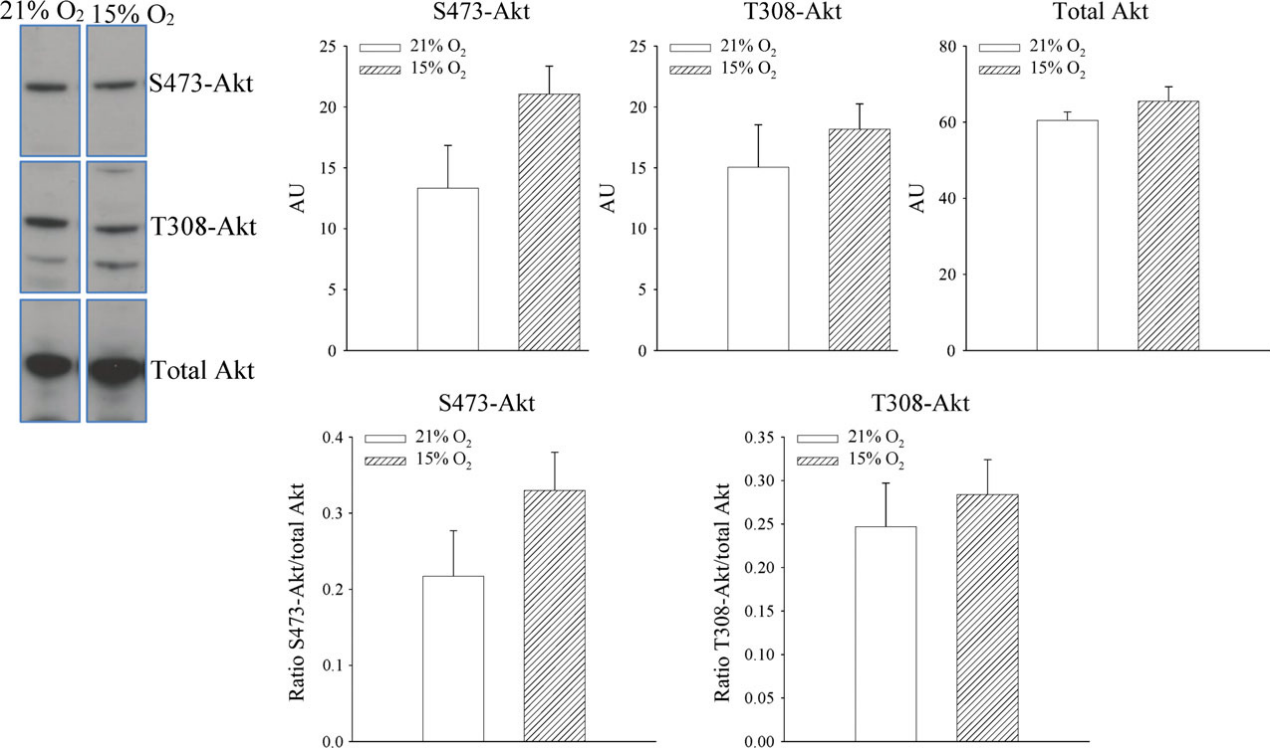
The effect of systemic exposure to 15% oxygen on phosphorylation of Akt in the myocardium. Original Western blots with phospho‐S473‐Akt, phospho‐T308‐Akt and total Akt antibodies applied on extracts from hearts under depicted conditions and corresponding graphs. Each bar is a mean ± S.E.M. (*n* = 4–5). *P < 0.05. AU: arbitrary units.

**Figure 4:**
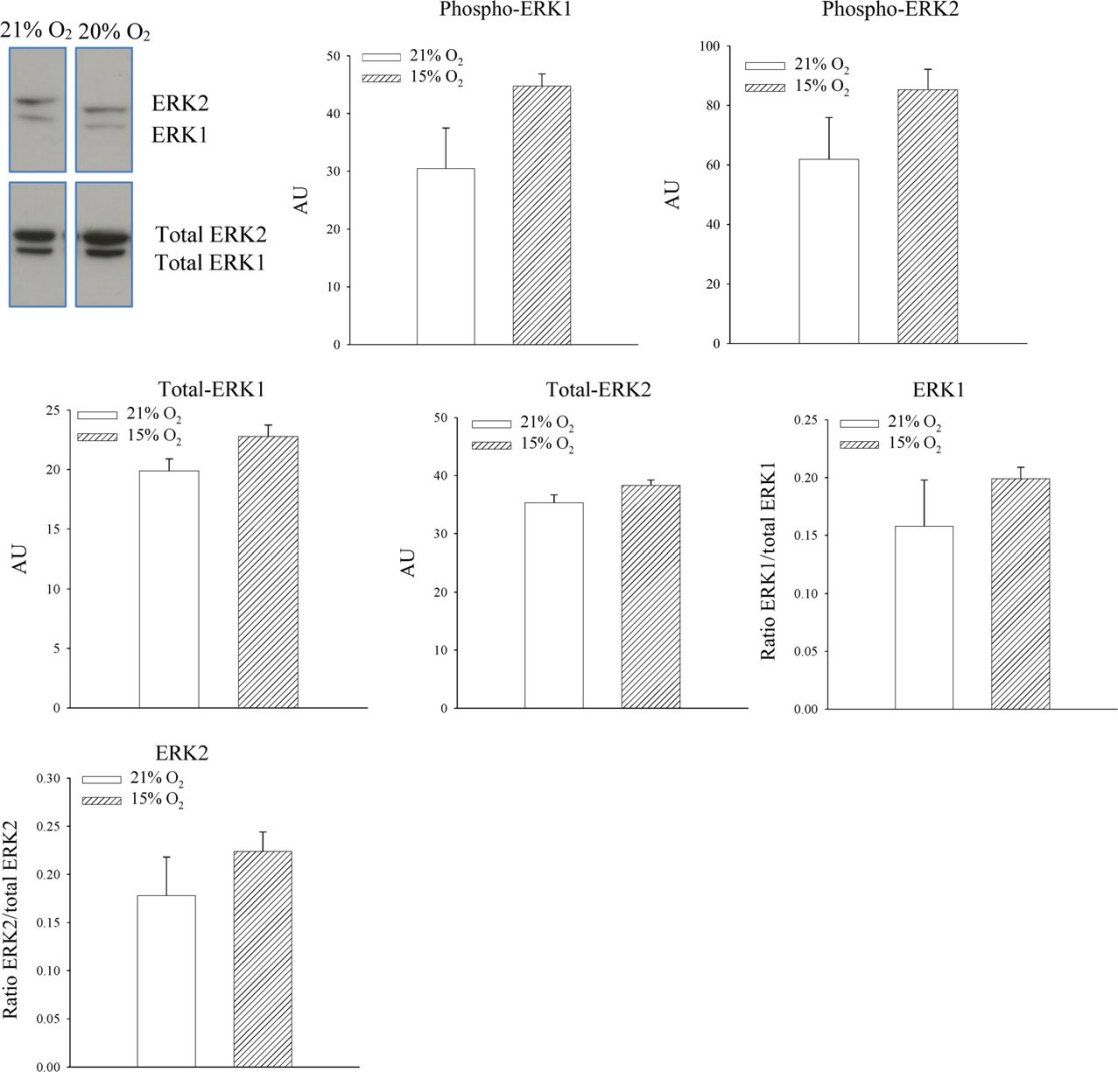
The effect of systemic exposure to 15% oxygen on phosphorylation of ERK1/2 in the myocardium. Original Western blots with phospho‐ERK1, phospho‐ERK2 and total ERK1 and ERK2 antibodies applied on extracts from hearts under depicted conditions and corresponding graphs. Each bar is a mean ± S.E.M. (*n* = 4–5). *P < 0.05. AU: arbitrary units.

An increase in SUR2A is known to induce cardioprotection 5, 6, 7, 8, 9, 10. PI3K‐Act is a cardioprotective signalling pathway, and from this perspective, it could regulate SUR2A expression. On the other hand, it has been shown that chronic mild hypoxia activates *in vitro* ERK1/2 leading to increase in SUR2A levels in heart embryonic H9c2 cells 21. Therefore, in this study, we have tested whether phosphorylation of Akt and/or ERK is activated by exposure of mice to 15% oxygen. Exposure to 15% oxygen was not associated with significant phosphorylation of S473 Akt and T308 sites (S473 Akt: signal intensity: 133.3 ± 35.1 AU in mice exposed to 21% oxygen and 210.6 ± 22.8 AU in mice exposed to 15% oxygen, *n* = 4–5, *P* = 0.083; Fig. 3; the ratio phospho‐S473 *versus* total Akt was 0.22 ± 0.06 for mice exposed to 21% oxygen and 0.33 ± 0.05 for mice exposed to 15% oxygen, *n* = 4–5, *P* = 0.082; Fig. 3; T308 Akt: signal intensity: 150.6 ± 34.8 AU in mice exposed to 21% oxygen and 181.8 ± 20.7 AU in mice exposed to 15% oxygen, *n* = 4–5, *P* = 0.427; Fig. 3; the ratio phospho‐T308 *versus* total Akt was 0.25 ± 0.05 for mice exposed to 21% oxygen and 0.28 ± 0.04 for mice exposed to 15% oxygen, *n* = 4–5, *P* = 0.585; Fig. 3). Exposure to 15% oxygen did not have any effect on either ERK1 or ERK2 (signal intensity: ERK1: 30.5 ± 7.0 AU in mice exposed to 21% oxygen and 44.8 ± 2.1 AU in mice exposed to 15% oxygen, *n* = 4–5, *P* = 0.382; ERK2: 61.9 ± 14.0 AU in mice exposed to 15% oxygen and 85.2 ± 6.9 AU in mice exposed to 21% oxygen, *n* = 4–5, *P* = 0.156; Fig. 4). The ratio phospho *versus* total ERK was also not affected for both ERK1 (0.16 ± 0.04 for mice exposed to 21% oxygen and 0.20 ± 0.01 for mice exposed to 15% oxygen, *n* = 4–5, *P* = 0.602; Fig. 4) and ERK2 (0.18 ± 0.04 for mice exposed to 21% oxygen to 0.22 ± 0.02 in mice exposed to 15% oxygen, *n* = 4–5, *P* = 0.344; Fig. 4).

### 24‐hrs‐long exposure to 15% oxygen induces AMPK phosphorylation

**Figure 5:**
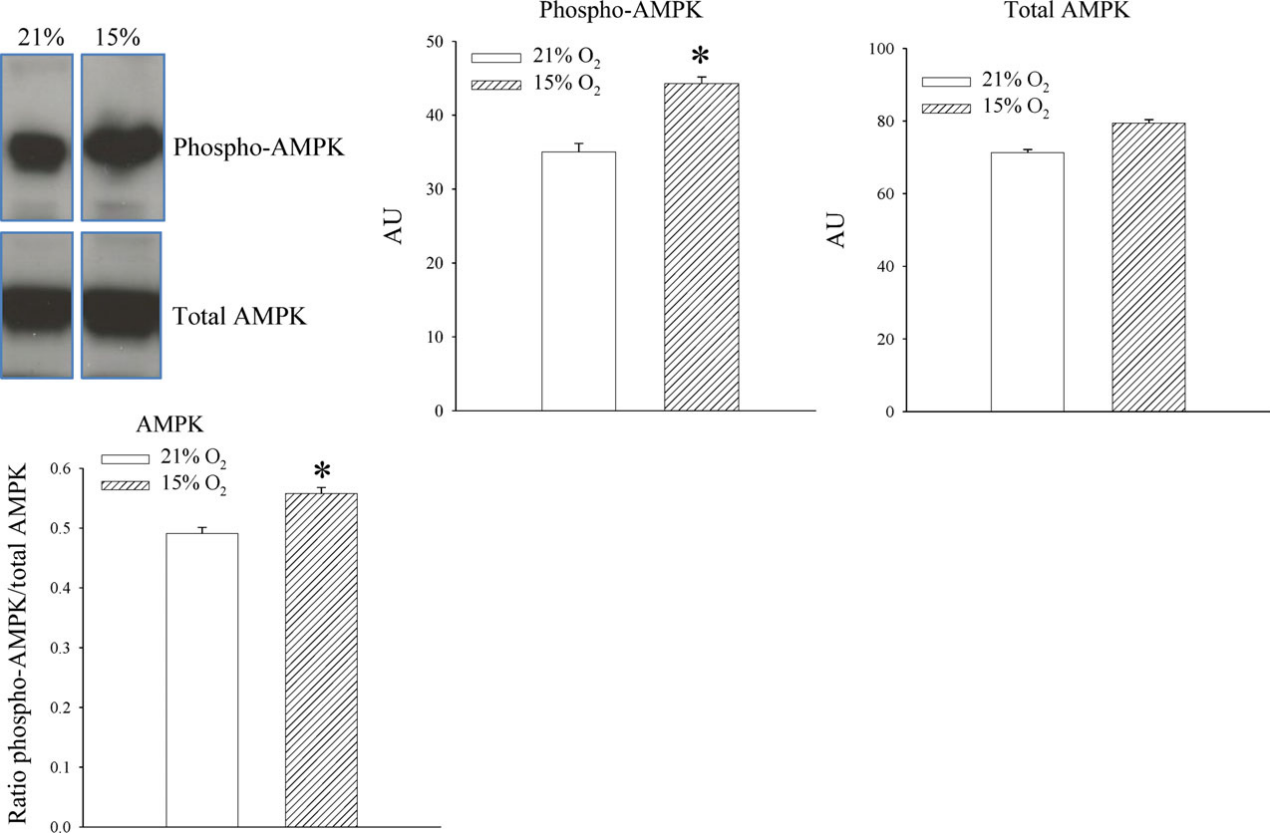
The effect of systemic exposure to 15% oxygen on phosphorylation of AMPK in the myocardium. Original Western blots with phospho‐AMPK and total AMPK antibodies applied on extracts from hearts under depicted conditions and corresponding graphs. Each bar is a mean ± S.E.M. (*n* = 4–5). AU: arbitrary units.

Activation of AMPK is known to stimulate trafficking of SUR2A and K_ATP_ channels 25. Therefore, we have examined whether exposure to 15% oxygen induces AMPK phosphorylation. A low but statistically significant increase in phosphorylated AMPK was observed (signal intensity: 35.0 ± 1.1 AU in mice exposed to 21% oxygen and 44.3 ± 0.9 AU in mice exposed to 15% oxygen, *n* = 4–5, *P* < 0.001; Fig. 5). The ratio phospho *versus* total AMPK was also significantly increased after animals exposure to 15% oxygen (ratio was 0.49 ± 0.01 for mice exposed to 21% oxygen and 0.56 ± 0.01 for mice exposed to 15% oxygen, *n* = 4–5, *P* = 0.002; Fig. 5).

### AICAR increases the level of SUR2A in embryonic heart H9c2 cells

**Figure 6:**
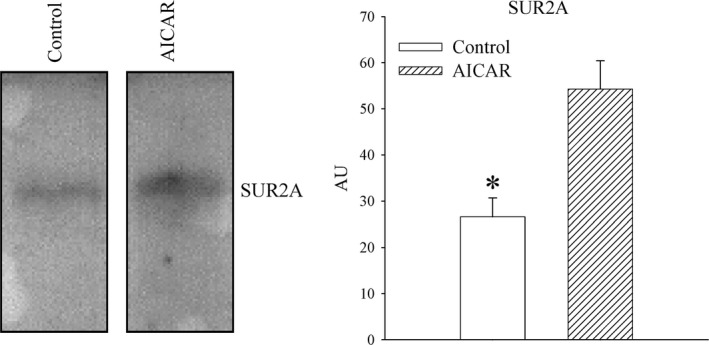
The effect of AICAR on SUR2A levels in H9c2 cells. Original Western blots and corresponding graphs under labelled conditions. Each bar is a mean ± S.E.M. (*n* = 4–5). *P < 0.05. AU: arbitrary units.

AICAR is a well‐established activator of AMPK 26. Here, we have exposed heart embryonic H9c2 cells to AICAR (1 mM) for 24 hrs. We found that AICAR increased the level of SUR2A in these cells (Fig. 6; signal intensity was 58.2 ± 4.8 AU in control cells and 85.6 ± 2.6 AU in AICAR‐treated cells, *n* = 4–5, *P* = 0.001).

## Discussion

Normobaric 15% oxygen corresponds to the oxygen tension at ~3000 m above sea level. In our previous studies, we have elucidated the regulation of SUR2A by other oxygen tensions. Specifically, we have examined the effect of 20% oxygen (350 m above sea level), 18% oxygen (1200 m above sea level) and 13% oxygen (3600 m above sea level). In all three oxygen tension conditions, mice exhibited increased SUR2A, but this occurred through distinct signalling cascades. For 20% oxygen, it was *via* ERK1/2 while for 18% oxygen, it was *via* PI3K/Akt 8, 22. In contrast, for 13% oxygen (severe hypoxia), no kinase was found to be phosphorylated and SUR2A levels were increased due to changes in intracellular ATP levels 9.

In the present study, we have found that exposure to 15% oxygen increased myocardial SUR2A levels in normal mice, but without activating ERK1/2 or PI3K/Akt signalling pathways. In mice exposed to 15% oxygen, PO_2_ in the blood was significantly lower than in mice at ambient oxygen, which is consistent with exposure to hypoxia 27. On the other hand, ATP and lactate levels in the heart were decreased and NAD+/NADH ratio increased. However, the difference was not statistically significant. It is generally accepted that hallmarks of myocardial hypoxia are decreased ATP, changes in NAD+/NADH ratio and increase in lactate 28. It seems logically to expect that more pronounced hypoxia would induce more pronounced decrease in ATP, an increase in lactate and changes in NAD+/NADH ratio. However, our findings are not in agreement with such notion. As an example, exposure of mice to 18% oxygen induced significant increase in lactate that was consequent to LDH up‐regulation 8. On the other hand, 13% oxygen induced an unexpected increase in myocardial ATP levels due to up‐regulation of creatine kinase 9. Thus, different oxygen tensions are associated with different patterns of gene expression and regulatory networks, which influence intracellular levels of metabolites in different ways. This could explain our findings that 15% oxygen induced less changes in intracellular lactate and NAD+/NADH than 18% oxygen 8. Also, 18% oxygen did not have any effect on intracellular ATP levels 8, while 15% oxygen had the tendency to decrease ATP albeit nonsignificantly. Accordingly, different metabolic response to different oxygen tensions could account for activation different signalling pathways under such conditions. Despite these differences in general response, 15% oxygen induced significant increase in SUR2A without affecting Kir6.2, which is similar to results reported in subhypoxia 19. It has been shown that SUR2A regulates the level of sarcolemmal K_ATP_ channels in the heart and that the level of SUR2A dictates the level of fully assembled channels 5. Thus, an increase in SUR2A results in increased number of sarcolemmal K_ATP_ channels, which, in turn, is cytoprotective 29. Such regulation of a single K_ATP_ channel subunit is not surprising when known that regulation of both subunits rarely happens 2, 6, 7, 8, 9, 30, 31.

It has been suggested that high‐altitude residents have lower mortality rates for ischaemic heart disease 16, 17, 18 and animal experimentation demonstrated that exposure to moderate hypoxia confers cardioprotection 19, 20. The underlying mechanism is complex and involves adaptive metabolic reorganization and metabolic gene remodelling 28. SUR2A has been recently shown to be a crucial regulator of myocardial resistance to different types of stresses, including ischaemia‐reperfusion 5, 6, 7, 8, 9. An increase in SUR2A, irrespective of what caused such increase, leads to increased myocardial resistance to stress 5, 6, 7, 8, 9, 10, 21, 22, 23, 29. In addition, it has been suggested that SUR2A controls cardiac ageing 10, 11. More specifically, it has been shown that ageing is associated with decrease in cardiac SUR2A levels and that maintaining normal levels counteracts ageing‐induced increase in myocardial susceptibility to stress 10, 11. Mice with nontargeted expression of SUR2A are characterized by increased physical endurance and slower decline in physical performance during ageing 7, 10, and they seem to live significantly longer than wild‐type mice (our unpublished observation). Specifically, we have left four male SUR2A mice (phenotype described in refs. 5, 6, 7) until their natural death. All mice lived considerable longer than expected and the shortest lifespan in this cohort of mice was 1124 days and the longest was 1270 days (average lifespan was 1182 ± 33 days, *n* = 4). Lifespan of mice can vary considerably depending upon a particular strain. Average lifespan of a male mice of a strain used to make SUR2A mice is 735 ± 17 days (*n* = 97, 23), which is significantly lower than those for SUR2A mice; an increase in SUR2A expression seems to increase lifespan for about 60%. SUR2A mice also lived significantly longer (23.6% longer) than C57BLKS/J mice that have the longest lifespan of usual mice laboratory strains (956 ± 18 days, *n* = 100, ref. 32). Thus, increase in SUR2A could account for prolonged lifespan reported in people living at high altitude 17. Thus, manipulation with SUR2A could be used as a therapeutic strategy against heart diseases and for purpose of prolonging lifespan. However, it should be pointed out that this is a preliminary study and more research on experimental models with cardiovascular pathologies should be performed before strong conclusions can be drawn.

Decrease in oxygen tension at various degrees is associated with up‐regulation of SUR2A, but different signalling cascades mediate up‐regulation of SUR2A at different oxygen tensions. About 20% oxygen was shown to activate ERK1/2, 18% oxygen activated PI3K/Akt and 13% oxygen did not activate any of these kinases while all three oxygen tensions did not activate AMPK 8, 9, 22. Here, exposure of mice to 15% oxygen did not affect ERK1/2 or PI3K/Akt, but it did activate AMPK. It has been recently shown that change in intracellular AMP/ATP is not required for activation of AMPK in hypoxia 33, which is in agreement with our findings that 15% of oxygen was not associated with significant changes in intracellular ATP. AMPK is known to mediate preconditioning‐induced cardioprotection by stimulating trafficking and activity of sarcolemmal K_ATP_ channels 25, but a link between SUR2A and AMPK has never been previously established. Therefore, to test whether activation of AMPK up‐regulates SUR2A, we have examined the effect of AICAR, a known AMPK activator on SUR2A expression in the embryonic heart H9c2 cell line. We have found that AICAR treatment increases the level of SUR2A in those cells, hence showing that AMPK activity regulates expression of SUR2A. Up‐regulation of SUR2A by AMPK is in agreement with previous work suggesting that chronic activation of AMPK is associated with cardioprotection 25. It is well established that hypoxia‐inducible factor (HIF) is the master regulator of the transcriptional response to hypoxia 34, 35. However, we have previously shown that HIF does not regulate SUR2A expression 21 and this is supported by our findings that even small changes in oxygen that do not activate HIF regulate SUR2A levels 22. The relevance of SUR2A in chronic adaptation to high altitude is yet to be fully established as we have here tested only the response of mice to 24‐hrs‐exposure to 15% oxygen. However, such relevance is likely as it has been shown that mild hypoxia *in vitro* increases SUR2A mRNA within 24 hrs and that longer stimulation of signalling pathways up‐regulating SUR2A results in sustained increase in SUR2A 6, 7. Nevertheless, future studies on regulation of SUR2A by chronichypoxia are required.

This study further adds to previous studies showing that different oxygen tensions activate distinct signalling cascades 5, 6, 19. However, irrespective of the cascade that is activated, the end result is always increased levels of SUR2A. It seems that SUR2A plays a central role in regulating myocardial resistance to hypoxia and oxidative stress and up‐regulation of this protein seems to be a compensatory mechanism in decreased oxygen tensions and oxidative stress of different magnitudes.

In conclusion, this study brings for the first time preclinical proof of the AMPK‐mediated regulation of SUR2A activation due to 24‐hrs exposure to 15% oxygen.

## Conflict of interest

The authors confirm that there are no conflict of interest.
